# Secondary students’ values and perceptions of science-related careers: responses to vignette-based scenarios

**DOI:** 10.1007/s43545-021-00130-9

**Published:** 2021-04-30

**Authors:** Keith S. Taber, Berry Billingsley, Fran Riga

**Affiliations:** 1 Faculty of Education, University of Cambridge, 184 Hills Road, Cambridge, UK; 2 School of Teacher Education and Development, Canterbury Christ Church University, Canterbury, UK; 3 Faculty of Education, University of Cambridge, Cambridge, UK

**Keywords:** Student perceptions of careers, Extra-scientific values, Ethics and science education, Cultural values and science education, Emotional responses to science

## Abstract

There has been concern about the attractiveness of science-based careers to many adolescent learners, and it has been suggested that school science may not always recognise or engage personal values that are important to young people in making life choices. The present study discusses interview comments made by upper secondary level students in England when 15 young people were asked to give their personal responses to brief vignettes describing scientific careers. Using an interview-about-scenarios approach, the students were asked about whether they would feel comfortable working in the scientific careers represented. The career areas were purposefully selected because they might be considered to potentially raise issues in relation to personal values or commitments that some students might hold. A range of student perceptions relating to the mooted careers were elicited (positive, negative and indifferent), but all of the participants raised issues that impacted on the acceptability or attractiveness of at least one of the mooted scientific careers, in terms of aspects of their own personal beliefs and values systems. It is recommended that teachers and career advisors should be aware of the range of value-related considerations that influence student views of science-related careers and should consider exploring aspects of science-based careers that link to values commonly shared by young people. This exploratory study also offers indications for directions for further research exploring how learners’ value systems impact upon their perceptions of science and scientific work.

## School science curriculum and extra-scientific values

There has been widespread concern about young people’s attitudes to science and science-related (or STEM, science-technology-engineering-mathematics) careers both in the UK (where the present study was carried out) and elsewhere (DeWitt et al. 2013). Amongst the factors that have been considered significant are students’ perceptions of their aptitude in areas of science; the assumed or perceived personality traits of scientists; perception of the gender appropriateness of careers; the perceived relative difficulty of STEM subjects and careers compared with other options; and responses to experience of school science as presented in the curriculum in terms such as enjoyment, interest and relevance (Krapp and Prenzel 2011). It is also recognised that learners may have limited impressions of what scientific careers involve, and they may be strongly influenced by input from significant adults (Aschbacher et al. 2010).

School science curriculum has itself been under a good deal of scrutiny internationally, prompted in part by issues of student experience and perceived relevance of school science (Stuckey et al. 2013). Recurring themes have been an emphasis on teaching for such notions as scientific literacy (Crowell and Schunn 2016), science for all (Millar and Osborne 1998), and science for citizenship (Evagorou and Dillon 2020; Sadler and Zeidler 2009) and epistemic insight into how science relates to religion in life's ‘Big Questions’ (Billingsley et al. 2020). Part of this development concerns a shift from learning science presented primarily from within disciplinary structures (where examples of applications are used to reinforce the principles and concepts that have been studied in their own terms), towards learning science within contexts considered accessible to students (such as food, clothing, transport) (Holbrook and Rannikmae 2017), and within discussion of socio-scientific issues (Sadler 2011) and multidisciplinary, real-world problems (Billingsley and Nassaji 2019).

Teaching about socio-scientific issues goes beyond contexts of applications (e.g. which would be the best material for preparing a clothing textile to meet particular specifications) to consider the complexity of real-world issues and scenarios—often those familiar to the students. One example concerns a unit looking at decision-making during an outbreak of SARS (Wong et al. 2011) which was developed in Hong Kong after a real-life outbreak which had been a major episode much covered by media. This engages what has been termed *informal* reasoning which “refers to the cognitive and affective processes involved in the negotiation and resolution of ill-structured issues and the rejection or adoption of positions or solutions” (Topçu et al. 2011, p. 314). The need for science education to engage such reasoning is being increasingly recognised in formal curriculum (Yap 2014).

A key aspect of teaching science *through socio-scientific issues* (rather than teaching *about applications*, or teaching *through contexts*) is the extent to which science is *insufficient* as a basis for solving problems and resolving disputes. Reaching a solution or decision in learning activities that engage with a socio-scientific issue do depend upon applying science knowledge, but application of scientific knowledge, though necessary, is in itself is insufficient—as judgements have to be made drawing upon extra-scientific considerations, in particular the application of values external to science itself, extra-scientific values.

### The nature of extra-scientific values

The term ‘extra-scientific’ does not necessarily imply something in opposition to, or inconsistent with science, but simply a consideration from outside of the domain of the natural sciences. So, in this paper, positions informed by ethical and aesthetic values, and emotional responses, would be considered extra-scientific, whereas a stance that learning more about the natural world is inherently a good thing, whilst reflecting a value judgement, would not be considered extra-scientific as this would widely be seen as an implicit value informing the practice of science (Allchin 1999), a ‘scientific value’. (Some other examples of scientific values are listed below.)

Judgements reached in discussing socio-scientific issues may depend upon extra-scientific values: examples might include aesthetic values invoked by some when objecting to the siting of wind farms; ethical values seen as relevant to the use of non-human organisms in medical research, or the use of culling on economic, or even conservation, grounds. In England, well publicised examples might be the culling of badgers supposedly to protect cattle herds, or the culling of deer to protect woodlands from excessive grazing. Science can provide evidence, and support arguments, about, for example, whether culling badgers is effective in combatting the spread of bovine tuberculosis (TB), but *not* about whether it is morally right to use such an approach to achieve desired ends (McCulloch and Reiss 2017) which would be a judgement informed by ethical values. A policy judgement about whether badgers should be culled in areas with high levels of bovine TB would therefore be *informed by* the scientific evidence, but would not be exclusively *determined* by scientific considerations.

An example which has recently been of global importance and seen as widely relevant would concern policy responses to the COVID-19 pandemic where there has not only been rapidly developing scientific evidence regarding such matters as disease transmission, risks to the infected, and efficacy of treatments, and indeed regarding such matters as potential effects on mental health of severe restrictions on activity, but also much discussion about related public policy issues where the scientific knowledge is inherently insufficient to determine policy. This would include, for example, questions about impinging on personal freedoms (for example, of people to have visitors in their own homes, or to congregate in large groups in public), and whether it is morally right to restrict the activity of groups at low risk of serious harm in order to protect those in the community considered to be at high risk. Science can provide guidance on the likely outcomes of particular policies, but cannot, for example, offer guidance to public authorities on how to balance lives saved by periods of closure of many businesses against the resulting economic costs.

### Values and career choice

Research into progression to science degree courses and careers has explored a range of issues such as gender, social class, ethnicity, home background, and personality (Archer et al. 2015; DeWitt and Archer 2015; DeWitt et al. 2013). There seems to have been limited research considering how values may be linked to student perceptions of science career routes. It has, however, been recognised that some aspects of science may be found unpleasant by some students—a study that found diverse levels of both interest and disgust in students dissecting pigs’ hearts in high school biology did highlight “the need for teachers to be aware of the potential for negative outcomes such that if initial disgust levels are high this may put a dampener on interest in the dissection and subsequent potential for learning” (Holstermann et al. 2012, p. 191). In that study, a gender difference was found, as “overall, girls expressed higher disgust sensitivity than boys” (p. 185).

It is reasonable to consider that value judgements are linked with career choices such as degree courses and areas of employment. For example, in one study it was found that “political views are predictors of major [subject of degree] choice, with more liberal students more likely to choose a non-science major” (Porter and Umbach 2006, p. 41). A study that examined undergraduate perceptions of careers found that STEM careers were considered to be less associated with “communal-goal [intimacy, affiliation, and altruism] endorsement” than non-STEM careers, and suggested that “if women perceive STEM as antithetical to highly valued goals, it is not surprising that even women talented in these areas might choose alternative career paths” (Diekman et al. 2010, p. 1956).

### Extra-scientific values and science education

Traditionally science teaching has focussed on the technical knowledge science can offer, rather than how applying this knowledge engages with individuals’ wider value systems. Science education has sought to reflect what might be called ‘scientific’ values such as the need to be open-minded and self-critical (Mulhall et al. 2017); the importance of honest publishing of results to share knowledge within the community; the exclusion of bias in studies; seeking objectivity such that in principle the results of studies are independent of the particular researchers carrying out the work; the search for coherence between different areas of science and the valuing of overarching ideas that can subsume different concepts. Increasingly, it is being argued that science teaching should also engage with extra-scientific (for example, ethical or cultural) values because it is recognised that in practice most people in society employ scientific knowledge in much wider contexts (e.g. in the context of consumer choice, lifestyle choices, healthcare decisions, activism, and voting in elections). It has been argued that there is a “need for more insight into students’ ways of using values and different types of knowledge in their argumentation and decision-making” (Kolstø 2006, p. 1690).

### Aim of the study

The present study is a small-scale attempt to find out something of the way such extra-scientific values might influence students’ perceptions of scientific careers. This exploratory study attempts to be neither comprehensive in terms of the range of scientific carers, nor to have surveyed a statistically representative sample of upper secondary school students in England. Rather the present study *tests the viability of eliciting value-informed impressions through the technique of presenting scenarios*.

The study explores a conjunction (personal values and career perceptions) that seems to have attracted limited attention from within science education. There has been discussion within science teaching about the use of animals for dissection (Oakley 2012), something that seems to motivate some students, but is found morally reprehensible by others (Randler et al. 2014). This could be considered a socio-scientific issue in its own right: there is a strong argument that dissection is valuable for learning anatomy and physiology and that alternatives (such as computer simulations) cannot provide as rich an educational experience; but, for many people animal life has inherent value and should be respected, and indeed some people believe animals have rights akin to human rights, making killing animals for educational purposes unacceptable (Regan 2005). Deciding whether science teachers should use animal dissection in teaching therefore involves value judgements informed by (extra-scientific) ethical values. Science can tell us about the level of pain likely to be experienced when an animal is killed for dissection—but science cannot tell us how much we should value the life that is being destroyed in relation to any potential educational gain.

In the present study, secondary age students were asked in interviews about how comfortable they might be in working in some particular scientific areas. The specific specialisms were selected because the researchers felt that each mooted career choice could potentially involve the kinds of extra-scientific values discussed above. That is, that although students could (and did) respond to a career in terms of their interest in the subject matter, or perceptions of their aptitude for that kind of work, there was also potential for personal (i.e. extra-scientific) values to influence their perceptions.

The research question addressed by this study is: What, if any, extra-scientific values that contribute to upper secondary school students’ perceptions of the desirability of scientific careers can be discerned from their responses to short vignettes of scientific work?

The potential of this research question to lead to the identification of such values depends upon both upper secondary school students’ perceptions of scientific careers being impinged upon by their extra-scientific values (which is a working assumption of the study), and that the somewhat novel data generation technique employed will act as a suitable probe for this. The study is not intended to be a representative or comprehensive survey of all extra-scientific considerations that may contribute to upper secondary school students’ perceptions of scientific careers, but rather a test of method.

## Background to the study

The present study derives from a larger project that had as its primary focus students’ perceptions of the relationship between science and religion. The Learning about Science and Religion (LASAR) project included a sequence of interviews carried out with students across a range of English secondary schools over a 3-year period. The appropriate ethical clearance procedures were followed at the two Universities where project investigators were employed. Permissions for carrying out the research had been obtained from the schools, the students, and, where the age of the interviewee indicated, the parents. It was decided that it might be useful to include a section on reactions to science careers during one round of interviews (carried out in 2012). All students were interviewed in their schools, at a time organised by the school, and in a location provided for that purpose by the school. One of us (FR) interviewed students and included the scenarios, or as many of them as possible, when time allowed. Data were included from 15 students, from across 6 schools.

**Fig. 1:**
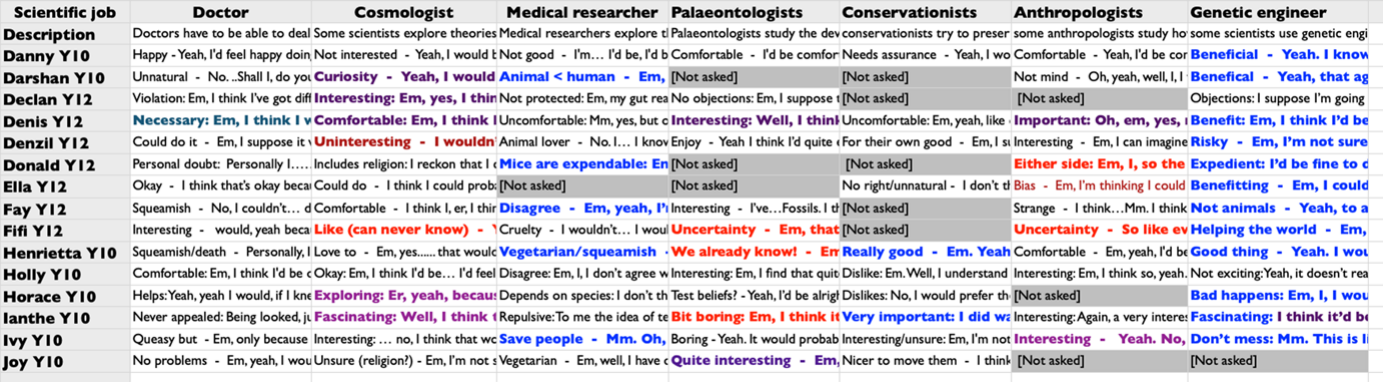
Image of spreadsheet used to summarise data

The 15 students were asked about five or more of these careers. (The number depended upon time constraints in the interview context—see Fig. 1). Eight of these students (Danny {asked about all 7 scenarios}, Darshan {5}, Henrietta {7}, Holly {7), Horace {6}, Ianthe {7}, Ivy {7}, and Joy {5}) were in Y10 {14–15-year-old students studying the compulsory school curriculum}) and seven (Declan {5}, Denis {7}, Denzil {7}, Donald {5}, Ella {5}, Fay {6}, and Fifi {6}) were in Y12 {16–17-year-old students having chosen to stay on at school for elective studies}).

These year groups represent the beginning of ‘key stages’ in the English school system (QCA 2004a). Students in Y10 will for the first time in their educational career have had the opportunity to make some choice about the studies studied in the school curriculum, although at this point are still required to study science. However, these learners will be aware that at the end of this 2-year (‘Key Stage 4’) phase of schooling they will both take high-stakes public examinations and be asked to consider more substantive educational career choices, which include the option not to continue with any formal study of science. The Y12 students in this study had selected academic subjects to study as part of academic courses leading to examinations that are used as the primary basis for admission to universities, as well as as key indicators for those entering other forms of education and training or employment. As the process of applying to University begins at the start of Y13, decisions about whether to apply, and for which subject, need to be made before entering Y13.

All the students included in this study had volunteered to be interviewed for the LASAR study. The initial letter of the assumed names used to ensure participant anonymity reflects the pseudonym used for the school in the study. Due to the practicalities of making school visits and arranging convenient interview times, Ella and Joy are the only participants in the present study from their particular schools, but there were six respondents included from one of the study schools.

## Interview context

Because of the main focus of the wider study, the interviews were organised around a semi-structured protocol including questions about the relationship between science and religion in general terms, about what science and religion suggested about a number of key issues (the origin of the universe, the origin of life on earth, prayer, miracles), and students’ experiences of how the theme of ‘science and religion' was treated in lessons (Taber et al. 2011). The English curriculum context at the time of the interviews included an expectation that the relationship between science and religion would be explored in lower secondary school (Billingsley et al. 2014), i.e. for students aged 11–14 (QCA 2004b), and that learning about science would include an appreciation of its cultural context (QCA 2007). This therefore provides the immediate context in which students were asked about their potential comfort with certain scientific careers. Undoubtedly, there is potential for what participants brought to mind when presented with our career-focused probes (the scenarios) to be influenced by the context of other issues that study participants had been asked to consider in the interviews. This does not undermine our contention that the data collected will have reflected genuine student values, but does need to be considered as a potential factor channelling their responses.

## Methodology

The overall approach used in the present study is a qualitative survey (Jansen 2010). Traditionally, survey methodology is considered nomothetic or ‘quantitative’ (Taber 2013), seeking to make statistical generalisations from a sample that is designed (or assumed) to represent a specific wider population. By contrast, qualitative surveys do not seek to establish frequency counts, from which statistical generalisations can be inferred, but rather seek to explore the variety of behaviours or (as here) cognitions amongst a sample through semi-structured interviews, and to describe the range of diversity found through themes or categories.

Ideally, we would have asked all interviewees about each scenario, however, in the event time pressures (for example, a student being due in a class) required making selections in some cases. However, each scenario was considered by at least ten participants (see Fig. 1), which we consider sufficient for the purposes of the study. Surveying a volunteer sample of 15 participants form across six schools was considered adequate as the study was considered as a test of method, rather than intended to offer either statistical findings or claims about an inclusive typology of considerations invoking extra-scientific values that might be identified in the wider population.

Interviews were used in our wider research programme as a complement to questionnaires. A questionnaire is a fixed form that presents items in a standardised way to different respondents, to support an assumption that responses can be aggregated and compared between sub-samples. By comparison, a semi-structured interview both admits incidental variation (the interviewer’s intonation and elocution is sure to drift; hearing conditions will vary in different schools; different interview locations will offer different distractions or stimuli) and opportunities for deliberate variations. For example, the interviewer can rephrase questions if considered necessary, can follow-up responses for clarifications or further explanations, can make decisions about changing sequences or, if indicated, omit items in response to the nature of participants’ contributions (Kvale 1996). The loss in standardisation is not considered inherently problematic when the foci of interviews, for example nuanced views about complex issues, indicates the need for an interactive data collection process. That is, this study is qualitative in the sense of requiring a researcher to use intersubjectivity as an instrument of research (Piantanida and Garman 2009). The admission of subjectivity (we cannot assume a different interviewer would have elicited *exactly* the same responses) is appropriate if we take an ideographic stance that values the position of individual respondents *qua individuals* rather than as simply part of a sample of a population (Taber 2013). Thus, the variation that might undermine rigour in a quantitative survey is seen as a potential strength in qualitative interviewing. This type of research looks for a depth of insight unlikely to be obtained through quantitative surveys, but with the proviso that *the participants cannot be considered to be representative of a wider population in any statistical sense*. The present study is, in that sense, a qualitative study, where limited weight should be given to the numbers of respondents who give particular kinds of responses to particular probes.

The actual data collection technique is a variation on the well-known technique of interviews-about-instances (White and Gunstone 1992), a method of elicitation which uses a series of probes that may be judged to relate to some focus (Gilbert et al. 1985) usually aimed at exploring student conceptual understanding of some topic—for example, forces, light, chemical bonding. This methodology has been extended to use scenarios as interview foci to elicit affective aspects of student thinking (Alsop and Watts 2000). In the present study, brief vignettes were devised to describe aspects of areas of scientific work. This study therefore seeks to extend the domain of application of the interviews-about-scenarios technique. Presenting a vignette of an area of scientific work posed a scenario inviting the participant to consider how they would feel about undertaking that type of work.

Here the probes are vignettes presented orally in the interview. The data collection technique used means that student responses are, in part, a reaction to the specific framing of the scenario (a point considered in the discussion later), but also draw upon any existing background knowledge they had about these areas of work. The vignettes were provided so that all students interviewed would have some basis for making a response, even if not already clear about the nature of the career. Whilst participant responses must be understood as arising in the context of the researchers’ particular characterisations of the careers (as in Table 1), this is in keeping with the notion that data arising in a qualitative interview is a co-construction between researchers and study participants (Kvale 1996).

**Table 1:** Scientific careers introduced through an ‘interview-about-scenario’ technique, and values expressed, and issues raised, by students in response to considering whether they might be comfortable working in those careers

Career option	Scenario	Values and issues raised
Medical doctor	Doctors have to be able to deal with very ill people, and sometimes with people in great pain or even dying. In their training they have to dissect human corpses to learn about anatomy. In their work they have to examine people with infectious diseases and, sometimes, horrible injuries	Worth of seeking to help others Squeamishness Dealing with death Life-and-death responsibility Sanctity of human bodies
Cosmologist	Some scientists explore theories of cosmology that try to find out about the origins and history of the universe. The working assumptions in this area are that the universe is thousands of millions of years old, and has slowly developed to have the structure astronomers see today	Lack of certainty of knowledge developed Potential for clashes with (or support for) religious beliefs Importance of basing thinking on evidence
Medical researchers	Medical researchers explore the nature of disease and the potential of different treatments to help cure disease or relieve pain and other symptoms. Sometimes medicines and treatments are tested out on non-human animals to see if they are effective. This involves giving animals diseases or injuries, and then comparing different treatments with the untreated animals. Sometimes these animals have to be killed and dissected so that the scientists can examine their internal organs	Can help people live and prosper Important to improve medical treatments Balancing numbers of lives sacrificed for numbers potentially saved Relative value of human and non-human animal lives Special status of humans Morally questionable actions Squeamishness Moral status of (non-human) animals Unfairness Undeservedness of human disease Animals cannot give consent Abuse of human power Relative value of animal lives of different species Specimens of abundant species valued less
Palaeontologist	Palaeontologists study the development of life on earth by examining fossils of living organisms that died a long time ago. These scientists work with the geologist’s models for how different rock formations were formed at various times in the last four thousand million years or so, and with the biologist’s model of how all the living forms on earth today evolved from the same very simple life forms which lived on earth over three thousand millions years ago	Limited scope to develop new scientific understandings Lack of certainty of knowledge developed Potential for clashes with religious beliefs
Conservationists	Conservationists try to preserve the different ecosystems on earth where different animals and plants are found. It is believed that many of the species on earth are in danger of extinction, and some times conservationists recommend killing some animals in certain places because there are too many for the food supply, or because one species (perhaps one not native to an area) threatens the existence of another	Good to put right human disruption of the natural order Existence of a preferred state of affairs that ‘should’ be Duty of care to maintain habitats Relative worth of lives of weak/sick and strong specimens Justification of sacrificing a few animals to save many Important to kill humanely Killing justified if animals breed quickly Relative value of animal lives of different species Identifying the interests of an individual animal (e.g. to be culled) with those of the wider population Animal species valued as sources of materials of use to humans
Anthropologists	Some anthropologists study how modern humans have evolved from other species over the last few million years. These scientists assume that modern human beings have been round for between a quarter and half a million years, and that their ancestors were physically different from people today, for example in the size and shape of their heads	Lack of certainty of knowledge developed Potential for clashes with religious beliefs But important to examine evidence Work could be biased by existing beliefs Uncomfortable thinking about having non-human ancestors
Genetic engineers	Some scientists use genetic engineering to produce new types of animals and plants. They take some of the genetic material from one type of living thing, and add it to a completely different type. This can, for example, produce crops which can better deal with pests or cold weather or lack of water	Value of addressing food shortages Value of improving efficiency of production and human (subsistence) incomes Value in improving nutritional value of crops Value in crops to replace non-renewable resources Value in developing strains to future-proof Human interference (meddling) in the natural order Purpose of genes is to allow evolution Potential for clashes with religious beliefs Modifying plants is a different matter to modifying (or cloning) animals Designer babies questionable Risks of unintended consequences Existence of a preferred state of affairs that ‘should’ be

The seven vignettes (presented in Table 1) concerned the following science-based careers:

- medical doctor;
- cosmologist;
- medical researcher;
- palaeontologist;
- conservationist;
- anthropologist;
- genetic engineer.

It is important to acknowledge that the choice of these particular careers (and how they were characterised) reflects, at least implicit, hypotheses on behalf of the researchers concerning potential issues that adolescent students might raise. Our original focus in the wider project had been student perceptions of science in relation to religion, but in this particular study we were interested in value-informed perspectives regardless of whether or not the participant considered these as related to religious beliefs. We are aware that the values found within a culture may be informed by religious traditions that have historically been influential in the culture, beyond those with committed religious beliefs, and exploring these connections was beyond this study: although some of the issues students raised (e.g. attitudes to human corpses, relative worth of humans and non-human animals, notions of how nature ‘should’ be) may reflect cultural norms and, in that respect, are certainly worthy candidates for further examination. The vignettes of scientific work (see Table 1) offered links to areas of science that some people find troublesome based on religious beliefs, such as human evolution, but we did not assume in advance that considerations identified would necessarily fall into this category. Issues that we were aware could arise included work that might link to scientific theories of origins which might seem to challenge some people’s religious beliefs (cosmologist, palaeontologist, anthropologist); attitudes to the human body, relating to such procedures as transplants or dissection or genetic medicine (medical doctor, medical researcher, genetic engineer); animal rights (medical researcher, conservationist, genetic engineer); and attitudes to humanity’s place in, and relationship to, nature (palaeontologist, conservationist, anthropologist, genetic engineer). It is clearly possible a different set of scientific careers or descriptions of them (just as a different sample of young people) may have elicited a somewhat different set of issues to those found in this study.

Collectively, we would consider that these scenarios potentially engaged with ‘extra-scientific’ values in the sense outlined earlier in the paper (see “Extra-scientific values and science education” section above), and so, in such cases, perspectives of scientific fields in relation to potential career choice can be considered a socio-scientific issue. As an example, medical research may inflect pain on non-human animals, and may require them to be ‘sacrificed’ for dissection. Science can suggest how much suffering is inflicted—though clearly no one can know for certain what an individual from a different species actually experiences (Nagel 1974)—and how many individuals of different species are killed, and can offer examples of the kinds of improved treatment and medical outcomes that such work has enabled. However, questions about whether it is right to use animals in this way, or how one balances the ‘cost’ to these animals against the potential benefits to humans in the future, cannot be answered from within science. Such issues may be nuanced: so, for example, it has been suggested that as non-human animals have no conception of their future (beyond the immediate situation) then killing them is not comparable to killing a person which robs that person of their chance to work towards a desired future. Science could potentially provide evidence for (or against) the hypothesis that only humans imagine, plan, and look forward to (or perhaps dread) the future; but whether deliberately killing a creature that has no conception of its future is morally justified is not a scientific question. Science has nothing to say about how we should value a potential future life that is totally unanticipated by an animal.

In some cases, the scenarios potentially link to extra-scientific values that might derive from religious beliefs (e.g. the fossil record can be understood in relation to both scientific accounts of natural selection as a contingent process, and faith-based accounts of the creation being purposive—and these considerations may potentially be perceived as complementary contributions to coming to an understanding, or as independent and unrelated, or as in tension). In the example just considered, of sacrificing animals in medical research, then questions of whether humans and animals are fundamentally different in terms of an immaterial soul; and of whether humans (i) stand in dominion over other species that have been provided to be used for human purposes, or, alternatively, (ii) have a duty of care to all living things as they are part of the same creation, may arise in, and be informed by, particular religious beliefs—but are extra-scientific.

However, extra-scientific questions of how we stand in relation to other types of living thing, or in relation to the wider biota, may also involve value judgements that are not necessarily *directly* derived from religious beliefs. As an example, scientists may potentially offer guidance on what level of species extinction is compatible with sustaining an environment suitable for human life, but a view that resources should be employed to minimise extinctions at a much lower rate than this may be formed based on valuing biodiversity as an inherent good, and this need not derive from any formal religious belief.

### Data analysis

Analysis of the data followed an approach consistent with the qualitative, idiographic nature of the data collection. Analysis was carried out by one of us (KST) who had devised the scenarios for the study. Qualitative data analysis usually involves several stages. There is usually a stage of preparing data for analysis; the breaking down of complex data into smaller units that can be examined individually (though with regard to the wider context); and a stage where the data are coded and/or categorised in some way. Depending on the purposes of the analysis and the methods employed, the initial codes/categories may then be used to build theory. In the extreme, in grounded theory research (Glaser and Strauss 1967), there is an iterative process of successively re-visiting analysis in moving from descriptive codes considered close to the data to more abstract theoretical codes as conceptualisation proceeds. More commonly, initial codes or categories may simply be grouped into higher order categories seen to reflect more general patterns across the dataset.

Research can be caricatured as following two broad patterns (Taber 2013): either primarily designed to test specific hypotheses, or more exploratory work seeking to discover new patterns. The former type is associated with a primarily deductive logic for data analysis, where codes and categories are largely predetermined in advance of collecting data (which may mean many of the data collected are not well described by the predetermined codes and so do not contribute to findings, something which is justified in responding to the specific research questions). The more exploratory mode tends to take a more inductive approach where the analyst seeks to identify the codes and categories that ‘best’ describe the dataset—however, with the provisos that this description is not entirely neutral but is motivated by the specific foci of the study; and that the process inevitably depends upon the fund of interpretive resources the particular analyst has available. The analytical process in the present study followed the more inductive modality suitable for exploratory research.

Transcripts were prepared from the original interview recordings. These were made verbatim, as far as possible, including hesitations. Relevant sections of the longer interview transcripts, that is the sections of the interviews where the scenarios were presented, were identified as the texts to make up the data corpus for this study. Each of these texts (i.e. the truncated individual transcripts) was initially considered individually. The comments relating to different science-related careers were copied into different cells of a spreadsheet organised by participant versus scenario (see Fig. 1).

**Fig. 2:**
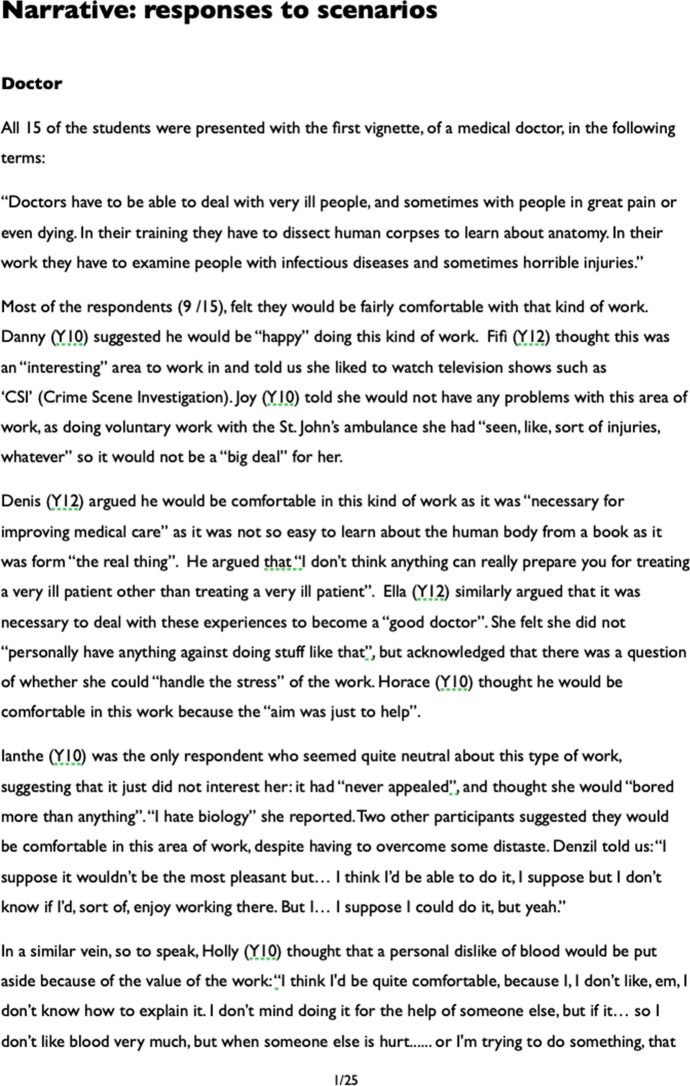
Image of the first page of the narrative produced as part of the analysis

Once this process had been completed, responses were considered by scenario, and a narrative document prepared using extensive quotations from the original comments, outlining the participants’ responses to the different careers (see Fig. 2). This type of approach to analysis has been labelled an ‘editing’ approach (Taber 2013, p. 299).

The narrative account produced at this stage was heavily based on the students’ own words and summarised all substantive responses available for each of the scenarios. This document was little more that 7000 words in length, and was then analysed thematically in terms of the core concerns of the present study to focus on where extra-scientific values were brought to the discussion. Consistent with the interpretative approach adopted in the study, the aim of analysis was not to produce ‘the’ definitive account of the data, as the substantive reduction of data inherent in such analysis necessarily involves a selection and foregrounding of what is perceived as most relevant to the researchers’ purposes, and relies on the intrinsically somewhat idiosyncratic interpretive resources available to the particular analyst. Rather, the intention is to produce an authentic account of the data in terms of the particular analytical focus or foci applied (here, extra-scientific values used to characterise scientific careers).

**Fig. 3:**
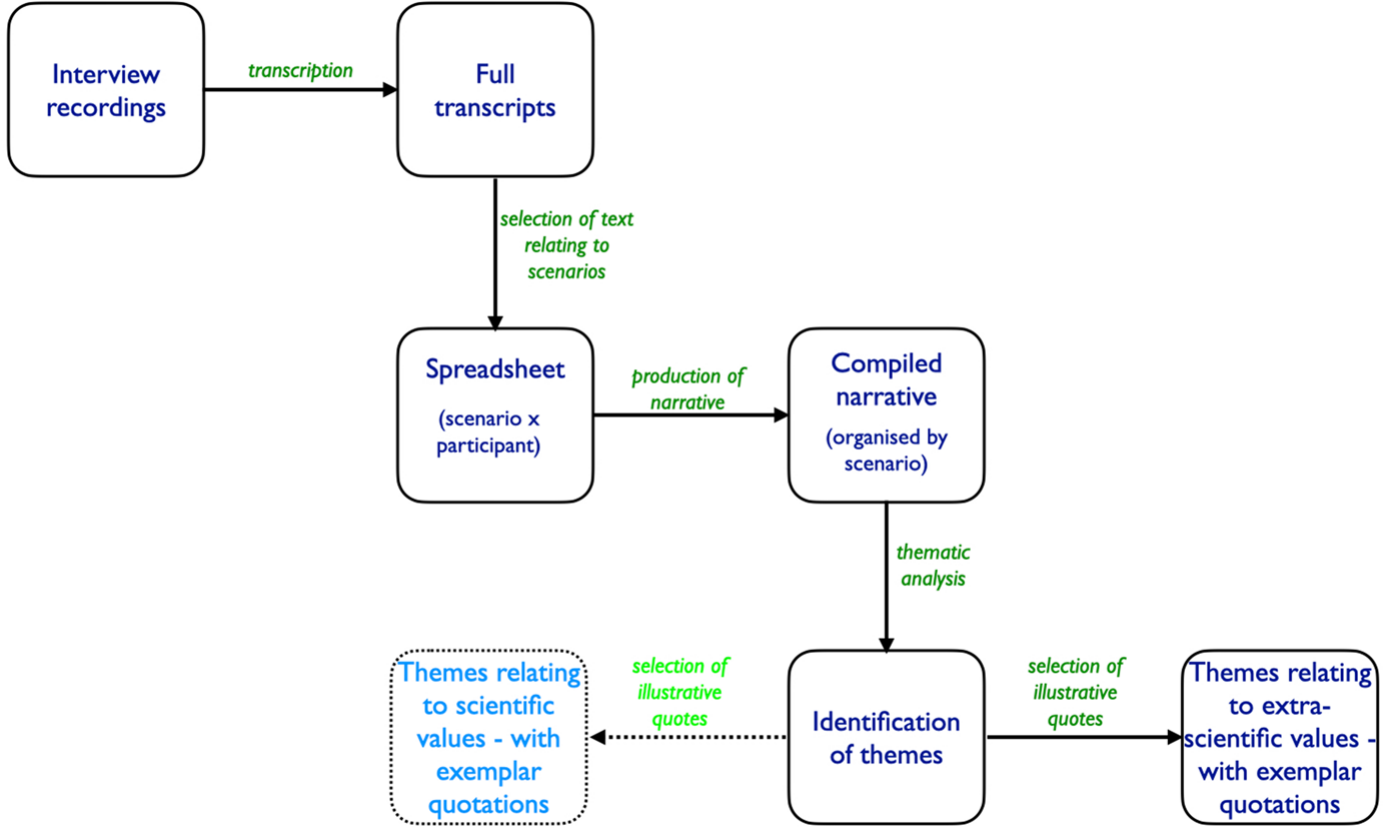
A schematic representation of the data analysis. This paper reports on themes related to extra-scientific values (themes relating to scientific values are reported in Taber et al. 2020)

The findings are reported below, arranged by scenario (i.e. area of scientific work), but organised into what were judged related comments. In our discussion section, we highlight some broader themes where extra-scientific values were invoked in responding to specific scenarios. It is important in qualitative work of this kind to offer readers support for the interpretations made by generous presentation of evidence from the source data; however, it is recognised that the normal conventions of journal reports limit the extent to which this is possible. A well-recognised researcher’s dilemma in representing data of this kind is finding a balance between the responsibility to reduce, analyse, and draw succinct conclusions from, qualitative data, whilst reflecting the complex nature of the source material and offering readers confidence in the analytical process (Pope and Denicolo 1986). The analysis presented in this paper draws upon the narrative account, which was itself too long to include in the paper. Speech is naturally different in form to the written word, and we have done some modest tidying of quotations (for example, removing some hesitations or repetitions) to aid readability—seeking to take care not to distort the intended meanings of our participants. The overall scheme for analysing the data is summarised in Fig. 3.

### Quality assurance and study limitations

The scenarios used in the study were prepared by the first author, who is a Chartered Scientist, and has experience of teaching science subjects to students in the relevant age range; and were checked by the other authors who also have school science teaching experience. We believe this offers some assurance of face validity of the instrument. By presenting the scenarios in full (see Table 1) those reading this report may also make their own evaluations. The students’ responses to the scenarios suggested they had no difficulty in making sense of them (engaging readily and responding directly without questioning or seeking further clarification).

The data were collected as part of an interview where rapport with the participants, all volunteers, had already been established. Students had been informed that they could ask to stop the interview at any time, or to decline to respond to particular items, but all participants responded readily to each scenario presented to them.

Interpretative research always relies upon an analyst interpreting the text produced by participants and clearly this process can never be absolutely assured. Here the analysis was undertaken in several stages, and we offer a full report of the process, and provide extensive verbatim extracts from the data, allowing readers to understand our procedures and make their own readings of the sample data.

The study is limited by its modest sample size (as is common in qualitative studies) and by the nature of the sampling. The selection of areas of scientific work was purposeful (as explained above) and so does not reflect all areas of the natural sciences. Additionally, some scenarios could not be presented to all participants because of time constraints (participants had timetabled school commitments to move onto). Moreover, the students are from a small number of schools, and had volunteered to be interviewed within the context of the theme of ‘science and religion’ and so cannot be assumed to be representative of the wider English school population at these ages. These circumstances are not problematic in relation to the purposes of the present study, but would need to be considered if any subsequent research seeks to either extend the present study to *comprehensively* survey where extra-scientific values may influence student perceptions of scientific careers or to *quantify* how frequently such extra-scientific values are relevant to student perceptions of scientific careers across the population.

## Findings

The focus here is identifying potential areas of concern learners may have about particular careers, in relation to their personal values, that might make them uncomfortable with the idea of doing that work (see Table 1). Some of the comments elicited from participants related to issues that linked to scientific values (as will be clear from some of the quotations below). In particular we recognised three themes. Two were that science offers powerful knowledge that allows people to change the world for the better, and that science supports a quest to answer the ‘big questions’ about the nature of our existence and place in the world. A third theme linked to the perceived relative value of knowledge in different scientific fields, in particular that in some (sic) fields it was not possible to obtain definitive, certain knowledge. Those themes are certainly of interest, and are reported elsewhere (Taber et al. 2020), whilst the present study is focussed on what we consider *extra-*scientific values. Participants also talked about whether they thought they would find careers interesting (or indeed boring), but, again, that is not a primary focus here.

### Doctor

Most of the respondents (9/15) felt they would be fairly comfortable with the work of a doctor, and appreciated the rationale behind such preparation for medicine as corpse dissection given that medicine was intended “to help” people (Horace). This (“when someone else is hurt”) might be sufficient reason to overcome a distaste, for example, of blood (Holly), although others cited being “queasy” (Ivy) or “squeamish” (Fay, Henrietta) as an obstacle. The perceived stressful nature of the work also offered a barrier (Ella), for example “working with people that are dying” (Fay), dealing “with the death” (Henrietta), and perhaps “this voice in the back of your head, what else could I have done to save them? … maybe I didn’t do it properly” (Donald).

However, two of the participants had principled concerns about human dissection, which did not “seem natural” (Darshan), or was even a violation (Declan):

…dissecting bodies, that’s one of the things that really put me off medicine. I was offered the chance to go around a dissection and as a gut reaction I said no. Because in a way you know that that person has gone, they haven't got any life, and you might say it’s just an empty vessel or body, but in a way something that still looks human in a way has a very human presence about it and it just feels wrong to be violating that presence.

### Cosmologist

Although the participants asked about being a cosmologist varied from considering it fascinating (Ianthe, Dennis) to being of little interest (Holly, Danny, Fay, Denzil), most suggested there was little about the career they would be uncomfortable about. Intriguingly, Denis referred to the “fascination of, you can see images through a telescope…of stars, for example, that aren’t actually there anymore…because the light has taken so long to reach it that the star has burnt down by now”, where, by contrast, for Denzil this made it “very old science”.

Four of the participants linked their responses to the potential connection between this area of science and religious beliefs. Only Joy seemed to be less comfortable with this area because of her religious beliefs, although she did not think that would “bother” her greatly, “because again I have my own opinions”. Declan suggested it was an interesting area, despite *not* illuminating the question of a creator God:

I think that’s a very interesting field because it is linking what we see now and trying to find the origin of it. It’s not saying we’re going to find out what started everything so that we can prove if there was a God that started it one way or the other. You know, that’s still very open for a religious mindset and for a scientific mindset.

Donald thought, in contrast, that cosmological research could support theological ideas as “it’s not just science, you can still bring in like religious concepts to it as well” and he gave the example that

it’s going to have, obviously, that overall scientific approach on like it was the big bang that caused it, but at the same time, starting millions of years ago well that’s sort of like God had to have created it to begin with, so something had to be in place there and religion and science overlap in that instance

Denis seemed to feel that the question of whether cosmological results would be consistent with religious beliefs was more open, but that this should not deter the work: as he “would want to find out the sort of the theory, with the most evidence behind it…rather than sticking fastly to one idea which I had”.

### Medical researcher

14 of the 15 participants (but not Ella) were asked whether they would be comfortable working in medical research. Two of our respondents were quite clear that they saw no reason to be uncomfortable in this area of research. Ivy remembered having discussed the issue of testing using animals in class, and told us that she “felt quite strongly that it should be used” for medical research “because only a few rats dying can save so many people”. She referred to the risks of instead testing medicines on people who were already seriously ill. She also volunteered that whilst being strongly in favour of medical testing on animals, this did not extend to testing cosmetics which was “a really big no”.

Donald, whilst acknowledging he would not feel “one hundred per cent comfortable”, pointed out that “by doing that you are then helping other humans”. Donald explained his position in some depth, valuing human life against expendable mice, and suggesting that there was something special about human life which was more than a product of natural selection:

…you might be harming mice but mice are widely abundant within this world. If the human population gets one disease and maybe that spreads to everywhere, and you don’t have that one overall cure, then you’re pretty much wiping humans off the face of the earth forever. Because if you take the religious point of view, then evolution doesn’t exist, humans won’t just keep popping up like that, it’s not like a chimp, every time I click my fingers a chimp becomes a human...so you need to do something to begin with to find all these cures and the fact that mice are so abundant, you’re not taking up the whole of the population to conduct an experiment, you’re taking a very small group out of the population to do these experiments on. And without that experimentation, then what would humans be like? We wouldn’t get all these cures that we have today that allow us to live through life and just prosper with it

Most of the respondents had greater reservations about medical experiments on animals. Danny felt he would be comfortable in medical research as although “it wouldn’t be good morally doing it to animals” this could be justified as “doing that would help other people”. Darshan had a similar perspective, feeling that “I think I would do that, but it’s not something I’d want to do”. He acknowledged it was “harsh” on the animals, but thought that “a human’s life…does come before an animal’s life…if you had the choice to save a human or an animal you would save the human, and so for the greater good of mankind, if you did discover a cure for a disease…by testing it on animals then you would”.

Declan, who had referred to *human* dissection as a violation (see above) told us he did not “oppose” medical animal testing “as much”, arguing that “while there is a very human presence even about a dead body, an animal because it doesn’t have…I don’t know, it may have the same senses as us, but because it doesn’t have the same communication with us, I don’t feel it’s, I don’t know, protected by moral laws”. The hesitations and qualifications here suggested that whilst Declan was firm in his conviction that “it feels much more of a violation to do something like that to a human than an animal”, he was perhaps less sure of his justification.

For a number of our participants, the case for medical research on animals was much less convincing. Denzil told us that “I know it’s necessary but I wouldn’t do it”. He explained that “I’ve grown up with animals and…[we are] sort of a family with animals and we are animal lovers so [I] wouldn’t do it”. Holly told us she would not be comfortable in that area of work. Although she felt that “they’re going to be getting something out of it” she could not see it was justified: “killing an animal just to find out why, and…I don’t, I just don’t…” Fay also told us “I don’t think I could do that…I personally would not be able to do it”, and seemed to be unsure on whether or not such work could be morally justified:

I think, this sounds awful...I don’t really agree with animal testing, but I think sometimes if it’s a really important cure for a human…you know, something for a really widespread disease or illness, and it could be solved by looking at a couple of animals, I don’t know what the right thing is to do, but I, I personally, couldn’t do that…Not me, no.

Denis felt he would not have been comfortable in that kind of work and explained that although

it’s very important to try and do things which will benefit medical treatments, but I think it really depends on where you stand on the relationship between humans and animals, whether they’re equal or whether humans would be given any kind of superiority. Even then I’m not sure if it’s…whether it’s right, in fact to kill animals…

Interestingly, three of our participants seemed to feel that although there was something inherently problematic with medical testing on animals, it might sometimes be justified. Henrietta told us she would “have an issue” with this area of work “because I'm vegetarian, and again I’m a bit squeamish”, however, despite this

I think medical research I could go into, because I think it is a really important thing that we try and find new treatments for things which kill humans which just isn’t fair, like…cancer. Nobody really deserves to die from something that is absolutely not their fault, there is no reason for them getting that, and it’s just a horrible thing. And it causes so much pain around the world. And so I think that’s a really good profession to be in, and I think I could do that. And I'd be fine with animal testing in theory, even if I couldn’t do it myself, because I can see the worth of human life over that of animals. That’s actually what my dad does.

Ianthe found “the idea of testing stuff on animals is utterly repulsive”. Despite this, she saw some medical research as being justified,

I have to accept that, for maybe from some medical reasons it’s necessary, you know finding a cure for cancer I have to accept is necessary, but like I’ve given up meat because I can’t contemplate just killing an animal for like what you want. You know, I think cosmetic animal research is disgusting. It’s really horrible and foul, and I don’t know how you could do that to an animal. Something that doesn’t have a choice in it. I think for medical research it’s very controversial. I think that possibly for like, there is a bit too much medical research on animals, but I think that it is necessary however horrible it is. But I just wish it wasn’t necessary.

Joy told us that she has “strong view on animals, like I’m a vegetarian” and that doing this kind of work herself “would really upset me”. As with Henrietta and Ianthe, though, Joy also considered the potential benefits of such work. Her thinking seemed to shift back and forth between a distaste for this type of research, and recognising its value:

I don’t like the thought of killing them and things like that. It just doesn’t seem right that just because we’re humans and we can, that we should. But I think a lot of the times with medicines they need to be tested. But I think it is slightly unfair that it’s done on animals because they can’t say that they don’t want it to happen to them. But then I think if we didn’t test it out on animals then we wouldn’t have medicines we do now, but then I don’t think I would be comfortable working…and doing that to the animals.

Two of our respondents felt it made a difference which species were being used in the work. So, Fifi told us that “I wouldn’t like to do that because of the fact that that’s cruelty to animals I think and…I think cruelty to animals is wrong because like they haven’t done anything”, yet:

on like rats and mice and things like that, I think it’s…okay because like you…kind of need to do it…Because otherwise how would we find out, but when they do it to like horses and dogs and rabbits and things like that, I don’t think that’s right.

She justified this distinction on the basis of how “rats and mice are, kind of, considered as like vermin”, but acknowledged that “I know other people have different opinions”. Similarly Horace thought “it depends what kind of animal it was” as he “would feel more comfortable doing it with a thing like a rat…because there’s a lot…[but] I wouldn’t do it to endangered species or anything like that”.

### Palaeontologist

Twelve of our interview participants (but not Darshan, Donald or Ella) were asked if they would be comfortable working as a palaeontologists. Most (9/12) of those asked thought there were no issues involved in this area of work to make them uncomfortable, although they had diverse views about the attractiveness of this line of work. Two of the participants in the study acknowledged that for some people this area of work had potential for clashing with religious beliefs. Declan noted that “I suppose this goes back to looking for the origins of life and sort of seeing the religious idea taken literally that things were there as they were, in science that they’ve developed”. Although he was not interested in working in this area, he told us “I wouldn’t have any, I don’t know, moral, spiritual objections to that, no”. Horace took a slightly different perspective, telling us that “I’d be alright going into that, just to see what my beliefs were and things like that”.

### Conservationists

Ten of our participants (but not Darshan, Declan, Donald, Fay, or Fifi) were asked whether they would be comfortable with the work of conservationists. Two of the participants seemed very positive about this area of work. Henrietta saw it as putting right damage people had done

I can see the worth of doing it, because – yeah, I think endangered species need to be protected. As humans we have destroyed a lot of the sort of natural balance of the world, and therefore it’s…really good for us to help put that balance a bit back in order. And if that does cost some animal life in order to preserve like animal life as a whole...then that’s acceptable...that job would just be kind of trying to put it back to how it should be....we’d be influencing it, but in a positive way to try and reverse the affect we’ve already had.

Ianthe took a similar stance, suggesting that despite some distaste for killing, a selective culling was on balance a positive act:

I did want to be a conservationist for about six years when I was a lot younger. I think it’s a very, very, very important bit of science. I think that we owe it every single animal out there to maintain their habitat because we’ve destroyed it so far. I can understand why culling some sort of animals is necessary. I think as long as they pick the weak ones. Like I have friends who shoot rabbits, but they only shoot the ill ones, and I think in many ways that’s beneficial. It’s hard, and I’m probably slightly hypocritical in that, you know…I oppose it so much, but I do think it’s slightly necessary. You know, fox hunting is something that I don’t like. I don’t like the idea of [killing for] pleasure, but I do understand that the ones that they kill tend to be the weak, the ill ones…I think that it’s better to give the stronger ones a better chance of health then, if in killing one you’re more likely to save ten. It’s kind of necessary, but it’s hard and I feel slightly hypocritical about it.

The other participants seemed to have stronger concerns or reservations about this area of work. Holly, whilst feeling she would not be comfortable in this work, was uncertain on the moral issues: “I understand why they’re doing it…but again, killing an animal…but then again, that is saving others, so I'm not really sure…I don’t know”. Danny seemed to accept the general principle of selective culling, raising the example of “the grey squirrels and…how…I don’t see any red squirrels anymore”, but questioned whether we could be sure of the effects of our interventions: “it’s killing one animal…is to do good by saving the other. But I'd have to have the knowledge of doing that, it has to be beneficial…”.

Denzil who had been uncomfortable with the notion of medical experiments on animals acknowledged that it might be “necessary” to sometimes cull animals, mentioning examples of deer and badgers, and justifying the killing as being a “quick death” that was “being done for a humane reason… And for their own benefit…you could, sort of, argue it’s for their own good”—although he acknowledged that evaluation might not apply to the *particular* animals that would be killed.

Ivy was less sure about this area of work suggesting “it’s selfish for humans to kill an animal just because they need more—more of something”, but accepted culling might be justified “if they’re damaging like…if it’s a necessity”. Joy also acknowledged that “would probably have to be done” to “rabbits and things like that because they just breed so fast”. However, she thought it would “probably be nicer for them to move them somewhere else”. Horace also suggested he “would prefer the idea of moving one batch of them to another place. I wouldn’t like killing quite a lot of them”.

Denis said that he would not “be terribly comfortable with killing animals”, and suggested that “you wouldn’t want to interfere too much in how things have happened for thousands of years, for example, even if it may result in the extinction of a certain animal”, although “there’s the problem of how that relates to humans, for example, if that one animal that becomes extinct is particularly of a particular use for human’s life, supply certain commodities maybe”.

Ella suggested interfering in nature was wrong, unless it was to undo human intervention:

I don’t think I could do that because unless the reason that they’re becoming extinct is due to human acts like, say tigers, they’re coming extinct because their environment’s been wiped out by people, by companies, wiping out wildlife. I could save them because it’s humans stepping in to something that they have no right to do, while something that was, occurring naturally like foxes eating rabbits and say rabbits were going extinct, I couldn’t do that because that’s just nature’s way of things happening and there’s nothing…we haven’t stepped in so far so there’s no reason to step in now.

### Anthropologist

Twelve of our participants (but not Delan, Horace or Joy) were asked whether they would be comfortable with working as an anthropologist. Several of our participants seemed to think there was no reason to be uncomfortable with this area of work (Danny, Darshan, Henrietta) often also noting it was an interesting field (Denzil, Holly, Ivy) even if not personally appealing (Ianthe). Denis who had suggested cosmology offered a context to compare scientific and religious thinking (see above), made a similar point in relation to anthropology:

I’d be comfortable doing that…I think I’m sort of more open to opinions, I would like to hold the sort of, you know, the opinion which has the most evidence behind it and I think studying anthropology is quite important when seeing where we’ve come from…em, alongside fields like biology and geology, so no I’d be comfortable with that.

Donald made a similar point, that “you’re just finding evidence to support either one claim or the other. If the evidence you find doesn’t support maybe the scientific claim, then it’s quite very likely that it will support the religion claim instead”. He said he was comfortable with this, even if “the outcome might not be what I think’s right” as “it will give like proper detailed answers onto like how the world was formed and like how our ancestors have evolved”.

Fay felt she might not be able to work as an anthropologist as she preferred not to think about *the implications* of human evolution:

I find that a bit, well a bit strange, the fact that we just look like we are now…I don’t know if I’d be quite comfortable thinking about it...the research side of it I wouldn’t mind, but then yeah thinking about it’s scary...Just the fact that we’re not, we’ve not always been humans.…it’s nicer to believe that we’ve always been like that because it’s quite strange to think that we descended from animals. It makes you feel not like you’ve always been human, I find that a bit odd.

### Genetic engineer

Fourteen of our participants (but not Joy) were asked if they would be comfortable working as a genetic engineer. Holly reported “I could do it” although “it doesn’t sound really exciting”. Most of our participants felt comfortable about genetic engineering and considered it had benefits for humanity. Darshan suggested that the work could be “enhancing” as “if you had crops…that could sustain and feed more people…I think that would be a very beneficial profession…to go into”. Henrietta suggested this area of work was

a positive thing, that like crops are influenced to make them, em, more effective because it just means less wastage, and for people in poorer countries who it is their livelihood to farm, then it’s a really good thing for them, because it just secures their income really, and it means they’re not going to be on the breadline.

Donald told us that “without doing things like that we might have a disaster in this world and then therefore the food supply might get cut off”, whereas

by developing processes where we know we can make crops, then we know that even if like the world changes so that the crops we currently have don’t grow, we might then be able to make these new ones, so we may not want to introduce them into our current food chains but we need to know the ways of making them so that if anything, God forbid, did happen, we have a way still of carrying on and still living.

Ianthe thought that this line of work would be “very, very, very interesting…fascinating” as well as leading to “a lot of like benefits”, and offered an example of “this woman in… South Africa, she was developing genetically modified maize that had many vitamins in that, you know…the current form of maize didn’t, and that people in poorer, hot, like dry countries could grow”. Ianthe was aware of “opposition” to this kind of science, and accepted it could be seen as “meddling” but thought it was justified “to give people a better life”. In a similar way, Denis acknowledged, but dismissed, the argument that “you’re interfering with the natural, the way things have been done for thousands of years previously” as genetic engineering has a “huge benefit” in relation to “world hunger” and he liked the idea of trying to “help out as many people as possible”. Danny took a similar view, suggesting that “generally religious people have a particular problem with it, they sometimes perceive it as playing God” but he took the view “it’s to better the…human race, and…I personally feel that it’s…beneficial”.

However, some of our participants did have some reservations about this area of work. Fay echoed the idea that “we should do it… if it’s going to help people, like the crop side of things” regardless of considering that “religious people would, you know, think it’s wrong messing around, and we shouldn’t mess around with these sort of things”. However, she limited this approval to working on plants, suggesting “when they mess around with animals and they make clones, so I don’t think that’s right…and also babies that are made to be genetic matches…I’m not sure about that either” so “if it was to go beyond plants…I would have a problem”. Ivy offered a similar view that “it’s different with animals…I think you have to be so careful”, but felt caution might be needed even working with plants. She thought that “it’s great you can get better crops” but “they have to be careful, because if you make them pest resistant, the pests become stronger…so it’s kind of like a cycle that you have to be careful with”. She suggested “don’t mess with nature too much”. Declan saw “more objections” with genetic engineering which was “trying to change and develop…what seems natural for us”.

Fifi thought this work would be “helping the world and the environment”. She offered a number of examples (“improving food sources and…saving the environment…how you can make, em, petrol and things like that from plants. And if you can get crops that grow faster…and… pest resistance and disease resistance”) although she also suggested there could be complications,

if they were resistant to one thing but then they were not resistant to this other thing then they’ll all get killed out, whereas if some of them were and some of them weren’t, some of it wouldn’t get killed off.

Ella told us “I could do that because it’s benefitting the survival of those plants” but seemed concerned about interfering with “nature’s way of dealing with everything”, as “it will help a lot of people but we can’t see what’s going to happen…like a lot down the line”. For example “it could eventually create something that’s poisonous but won’t be poisonous for a few more generations of plant”.

Denzil could “understand the benefits and I’m quite happy with them doing that”. He was aware that “a lot of people have, sort of, moral objections to it” but his own reservations concerned the work being “very risky” as “you do experiments and it can go wrong and could cause quite major problems…it could do more harm than good”. Horace also thought that he “wouldn’t really be that comfortable, because I don’t like to mess with genetics of things, because they’re, it’s just there so they can evolve”. He suggested that “bad things can happen if you mess with DNA, so I wouldn’t like some sort of super bug or something to come out”. He suggested “I just want that to stay there as it is, because it’s meant to be like that”.

## Discussion

We set out to explore whether is it possible to identify extra-scientific values that contribute to upper secondary school students’ perceptions of the desirability of scientific careers through responses to scenarios comprising short vignettes of scientific work. The scientific fields presented and summarised in the vignettes were purposefully selected because we considered they might raise ethical, or other value-related, issues for some students. Our participants engaged with the scenarios, and presented a number of views infused by extra-scientific values such as ethical or aesthetic values. Our vignettes could be criticised as having been designed to hint at potential issues, so, for example, conservationists might feel that the reference to culling animals was given undue weight in our vignette. However, our purpose was to test out whether a simple technique could elicit whether students' personal values might make some scientific careers seem less attractive, and the vignettes were designed to highlight features where some potential issues could arise.

The participants in this study offered a wide range of views on the scientific careers we probed them about. It was notable that, in this particular sample at least, there was often a recognition of the potential value of scientific work (including the sometimes controversial area of genetic engineering), and a view that scientific work was often interesting. There was also some squeamishness which made some occupations (practice and research in medicine) seem unattractive to some students *personally* even when they appreciated the value of the work. In the case of medicine, the sense of personal responsibility for literally life-and-death decisions also made the occupation unattractive to some of the students interviewed. As well as such issues of personal aptitude, the interviews revealed a range of other considerations linked to values that were revealed in considering the occupations discussed.

### Students’ values and perceptions of scientific careers

Although our participants often told us whether particular scenarios related to careers they might find attractive, this judgement could be informed by a range of factors such as interest in a topic and the perceived importance of the work. Judgements could also be informed by considerations related to aesthetic values—such as finding some aspects of medical work unpleasant. Perhaps, in some cases, judgements of what is interesting may have been influenced by earlier aesthetic responses to aspects of nature (fossils, the night sky, crystals) even if that is not explicit in a person’s current thinking. It seems unlikely that a technique such as employed in this study could disentangle student responses that may be informed by a long developmental history. For example, Fifi’s differentiation between the killing of rats and mice on the one hand and horses, dogs and rabbits on the other was presented in terms of what was ‘right’ (a moral judgement linked to ethical values) but may well have been influenced by aesthetic considerations that may at least in part themselves have developed from social norms in terms of how animals are widely discussed in the culture. For example, young children may learn that rats are ‘dirty’ but rabbits are ‘cuddly’. The scope of the present study does not allow moving beyond speculation in this regard. However, whatever their genetic origin, our method clearly elicited perceptions informed by both ethical values—relating to what is considered natural, the acceptability of killing, and the differential worth of different species—and by what can be considered epistemic values.

### Epistemic values

Earlier in this paper we gave some examples of what would be considered scientific values, and these might often be considered epistemic values as they are linked to the generation of scientific knowledge—relating to matters such as objectivity, and the publication of results. One example was the search for coherence between different areas of science, and in our study it was suggested that there was particular value to combining research from different scientific fields (anthropology alongside biology and geology—Denis) which could collectively offer unifying knowledge (something that might then be considered a scientific value).

Some other comments made by our participants can be considered to reflect epistemic values that can be considered to demonstrate alternative conceptions of the nature of scientific knowledge (Taber et al. 2020). Some of our participants commented on some scientific careers as being of less value either because they could not produce definitive, certain knowledge (cosmology, palaeontology, and anthropology for Fifi), or conversely, because they were primarily confirming already established knowledge (palaeontology for Henrietta). One participant thought she was unsuitable to work in anthropology because her existing beliefs about the field would bias her (here, to expect and look for links between fossils)—something that a modern perspective on the nature of science suggests would be normal (Lakatos 1970), if not inevitable (Kuhn 1970).

Students are expected to learn that (all) science only produces provisional knowledge, but it is common for students to consider science as proceeding from a scientist’s guess to certain knowledge (Driver et al. 1996), something that was reflected in our wider project (Taber et al. 2015). Whilst it is usually the case that originality is indeed valued in science (as elsewhere), replication of results is also valued as an important part of the process of knowledge generation. Where our participants considered fields to ‘only’ generate uncertain knowledge this could *also* raise issues where ethical values were invoked. So, some participants considered the work of conservationists (Danny, Denis) or genetic engineers (Ivy, Fifi, Ella, Denzil, Horace) to be potentially problematic because scientists’ actions could have unforeseen outcomes.

Another epistemic value seemed to be reflected in Denis’s comment about the importance of understanding our (human) origins, although interestingly Fay felt this kind of knowledge could be uncomfortable as it raised (“scary”) questions about the nature of being human. Although this sample of learners generally did not refer to religious beliefs as a potential barrier to entering scientific fields, there were some comments along the line that some areas of scientific research *could* support or contradict religious beliefs (e.g. “if you take the religious point of view, then evolution doesn’t exist”). This reflects other findings from the wider LASAR project that suggests that although the most common religious affiliations of people in England are to Christian Churches that do not see scientific accounts as a challenge to religious beliefs, many young people themselves assume that scientific accounts of origins (of the Universe, of life) are contrary to religious teaching (Taber et al. 2011). There were several points where our participants indicated that even if scientific work might lead to findings that seem to clash with their religious beliefs, it would be better to know this and engage with the evidence (which could be seen as reflecting a scientific mindset).

### Perceptions of the natural order

A range of participant comments linked to notions about *what is natural*, and actions which contravene a sense of natural order or law (which involves seeing some human actions in the world as in a sense outside of nature). So dissection of human corpses was seen as questionable by Darshan and Declan and some of our participants saw genetic engineering as a kind of meddling in nature, which could be morally questionable (Fay and Ivy)—even *if* this might sometimes be justified (Ianthe, Dennis and Danny). Ella thought some conservationist activity could be considered as interference in nature, although where human activity had already impacted on other species, Ianthe and Ella felt there was an obligation to work to put this right, and Henrietta implied there was a duty to protect endangered species and restore a natural equilibrium. Henreitta referred to a natural balance in the world that “should” be maintained, and Denzil implied that the current state of an organism’s genome is how it is “meant to be”.

### Moral questions about killing

Work that involved killing animals was seen to be questionable (or worst) morally (Danny, Denis, Holly, Ianthe, Ivy), and alternatives were to be be preferred—such as moving animals rather than culling them in conservation work (Joy, Horace). Ianthe and Joy both raised the issue of animals being unable to give consent to being sacrificed for research. This is not an issue usually considered by scientists as it is widely considered non-human animals are not able to have a conception of consent, or indeed even a conception of any desired future that their death would deny them. Denzil thought any killing should be humane. A utilitarian argument was offered that it is acceptable to kill a few members of a species (Denzil), especially the weak or diseased (Ianthe), to potentially save many conspecifics.

### Valuing of different species

Denis pointed out that judgements about the acceptability of killing animals in medical research depended upon considerations of whether (non-human) animals had similar worth to humans. However, Declan did not think non-humans were protected by moral law and a number of participants (Ivy, Donald, Darshan, Fay, Henrietta) thought it was clear that human life was to be valued more highly, and this justified killing (non-human) animals. This might be seen as a kind of utilitarian judgement of the acceptability of the loss of less valued lives when justified by the saving of more valued lives. This might be compounded when fewer members of a less valuable species might be sacrificed to save greater numbers of the more highly valued species (Ivy, Fay)—perhaps even when the species were as closely related as red and grey squirrels (Danny). It might also be seen as justified to kill members of a species judged to themselves be damaging (Ivy). It was also suggested that it was more acceptable to kill members of some non-human species for human benefit than others (Fifi, Horace). It was also notable that some participants felt that genetically modifying species was a more problematic issue when the species were animals than when they were plants. This seemed to be a strong intuition which may be worthy of further exploration in future work.

### Implications

The present qualitative survey is exploratory, seeking views on a select range of areas of scientific employment from a modest number of upper secondary students from a small sample of English schools. The purpose of our study was to explore how students’ values might influence their thinking about the attractiveness of different areas of science and to test the use of interviews-about-scenarios in this context. We sought to answer the research question: ‘What, if any, extra-scientific values that contribute to upper secondary school students’ perceptions of the desirability of scientific careers can be discerned from their responses to short vignettes of scientific work?’ It was found that although some of our scenarios cued reservations related to the students’ ethical, aesthetic, or epistemic, values such that sometimes participants suggested they would be uncomfortable in some areas of scientific work, there was also much evidence of the participants valuing scientific work both on the grounds of it providing knowledge to be valued for its own sake, and more especially that it could make a practical difference in meeting human needs or addressing environmental concerns (Taber et al. 2020). Even given the deliberate selection of scientific fields and the framing of scenarios through the specific vignettes, participants tended on balance to be pro-science. However, it has been previously pointed out that student attitudes to science itself (as understood through media, or personal contacts, such as Henreitta’s father working in medical research) may be quite different to attitudes to their experiences of school science (Osborne et al. 2003).

As a small-scale study, drawing on a sample of students who had originally volunteered to be interviewed around the broader theme of the relationship of science and religion, this research cannot offer meaningful indicators of the *proportions* of school age students who might share the views reported here—that would need a quantitative survey approach applied to a representative sample of learners. However, it is notable that these young people were generally engaged by being presented with our scenarios, and their readiness to speak eloquently about such issues such as the correct treatment of the dead, what should be considered ‘natural’, and when it is acceptable to kill non-human animals, suggested that these were matters they had given some thought to, and which did colour their thinking about their own future paths.

There has been much research recently about factors that engage students in studying science studies and encourage them to consider science careers (Archer et al. 2010). The present study tentatively suggests both that students may be engaged by more detailed consideration of the ethical and aesthetic aspects of scientific work, and also that further studies into how learners bring, and apply, their own value systems to thinking about the practices of science may be productive in understanding student thinking about possible careers, and in informing the selection and framing of curriculum material.

The study then raises issues relating to curriculum and pedagogy. Some of the epistemological assumptions revealed (about the relationship between scientific claims and religious beliefs; about some (sic) sciences producing uncertain knowledge; and others merely accumulating confirmatory evidence) suggest there is more work to be done in teaching about the nature of science. The issues that engage students suggest some themes for exploration in science classes that may act as contexts for introducing scientific ideas: e.g. how do we decide when a group of animals are vermin; what might be the costs and implications of seeking to move unwanted creatures rather than culling them? There is also much scope here for offering contexts for exploring issues that may invoke ethical values:

- does an argument that it is better to “*save* a human [rather than] an animal” imply it is acceptable to *kill* the [non-human] animal to save the human [animal]—given research that shows people are more likely to suggest they would make decisions based on consequentialist thinking when making choices over who to save, rather than when needing to kill someone in order to save others (Kamm 2015)?
- how can we justify killing some individuals to save their con-specifics?
- is the concept of consent meaningful outside of humanity?
- why are plants not deserving the same considerations as animals?

The application of science always raises extra-scientific considerations, including a degree of uncertainly about potential unintended consequences. For those students who may go into careers in science, these issues will need to be considered. The major areas of involvement in science for those who do not, but who will be citizens in democratic societies, will likely be with issues of the application of science (in consumer choices and personal behaviour; in selecting medical treatment; in public policy debates, engagement with pressure groups, and voting). There is clearly an argument here for giving higher priority to teaching about socio-scientific issues within the curriculum.

This exploratory study suggests there is much scope for more detailed research into students’ thinking about the nature of scientific work, alongside other strands of enquiry asking about learners’ science career choices. It suggests that students are generally supportive of science, but recognise a range of reservations about some types of scientific work. Moreover, if this sample of students is typical, then socio-scientific issues, and ethical questions arising in scientific work, offer strong foci for engaging learners in learning about science.

## Data Availability

The data used in this study are interview transcripts from conversational qualitative interviews which we do not have participant permission to share, and which if made publicly available might undermine the anonymity assured to study participants.
